# Vesicles-mediated resistance to antibiotics in bacteria

**DOI:** 10.3389/fmicb.2015.00758

**Published:** 2015-07-23

**Authors:** Madhab K. Chattopadhyay, Medicharla V. Jagannadham

**Affiliations:** Centre for Cellular and Molecular Biology (CSIR)Hyderabad, India

**Keywords:** antibiotic-resistance, outer membrane vesicles, OMVs, β-lactamases, membrane-active antibiotics, fluoroquinolones, carbapenems, horizontal gene transfer

## Introduction

During the past few decades, antibiotic-resistance of bacteria has assumed the proportion of a global crisis. The discovery of new antibiotics is not matching the rate of emergence of resistant strains thus narrowing the scope of chemotherapy. Notwithstanding the fact that emergence of resistance is facilitated by the use of antibiotics (Levy, 2002) exposure to antibiotics is not a pre-requisite for it. Antibiotic-resistance is detected even in bacteria occurring in places detached from the human civilization for millions of years (Bhullar et al., 2012). The various mechanisms responsible for antibiotic-resistance of bacteria were discussed in this journal some time back (Lin et al., 2015). Outer Membrane Vesicles (OMVs) are spherical bag-like structures (20–300 nm) released predominantly by gram-negative bacteria in the outer environment. They help the producer cells in communication with other cells, secretion, pathogenesis, acquisition of nutrients, and self-defense (Kulkarni and Jagannadham, 2014). Recent evidences indicate that they protect bacteria not only from phages and various environmental stress factors but also some antibiotics. The nature of vesicle-mediated antibiotic-resistance, known so far, is dealt with in this article.

## Involvement of the vesicles in antibiotic resistance

The OMVs mediate antibiotic resistance in various ways. In some cases they serve as vehicle for the transport of antibiotic-inactivating enzyme. Using polyclonal antibodies against chromosomal β-lactamase of *P. aeruginosa* and protein A-gold (Ciofu et al., 2000), demonstrated the presence of β-lactamase in OMVs isolated from three β-lactam-resistant clinical isolates and one β-lactam-sensitive strain of the bacterium. Gentamicin-induced increase in the vesicle formation by the same organism was evidenced earlier (Kadurugamuwa and Beveridge, 1997). Thus it was indicated that coupling of an aminoglycoside with a β-lactam antibiotic for the treatment of cystic fibrosis patients might be counter-productive. Imipenem-induced stimulation in vesicle formation has been recently demonstrated in the nosocomial pathogen *Stenotrophomonas maltophilia*. The vesicles were found to contain both L1 metallo-β-lactamase and L2 serine-β-lactamase (Devos et al., 2015). Available evidences also indicate that vesicles containing β-lactamase confer protection not only to the producer organism but also to some other bacteria co-occurring with the producer in the human respiratory tract (Schaar et al., 2011, 2014). Recently, it has been demonstrated that the β-lactamase associated with the vesicles produced by some members of the Bacteroides spp protected some commensal and enteric pathogens (e.g., *Salmonella typhimurium*) from the third generation cephalosporin cefotaxime. Species belonging to Bacteroides constitute a major fraction of the human intestinal flora. Hence the clinical significance of this observation needs hardly to be over-emphasized (Stentz et al., 2015). Bla Z, a β-lactamase protein was found to be associated with the vesicles produced by a gram-positive organism, *Staphylococcus aureus*. Survival of some ampicillin-sensitive gram-positive and gram-negative organisms incubated with the antibiotic was found to be mediated by the vesicles (Lee et al., 2013). These findings underscore the importance of OMVs in promoting emergence of resistant strains out of a mixed community of bacteria. Immunoglobulin G (IgG), produced against β-lactamase, is believed to inhibit the enzyme and thus to render β-lactamase-positive bacteria susceptible to the antibiotic. OMVs were found to protect β-lactamase produced by *Moraxella catarrhalis* from inactivation by anti-β-lactamase antibody by packaging the enzyme (Schaar et al., 2013). It appears that the β-lactamases could be packaged into the vesicles during the formation of the OMVs because of their location in the periplasmic space of bacteria.

It was observed that the growth inhibitory effect of colistin and melittin, two membrane-active antibiotics on an Antarctic bacterium *Pseudomoas syringae* Lz4W, was reversed by the addition of OMVs produced by the same organism, to the culture medium. The binding of the antibiotics by the vesicles was demonstrated by NPN-uptake assay. Proteomic analysis of the vesicles revealed the presence of proteases and peptidases in the OMVs, but no evidence of degradation of the two peptide antibiotics was obtained. This it was evident that the vesicles protected the bacterium by binding and removing the antibiotics from the extracellular milieu. The protective effect of the vesicles on the organism however was not observed against streptomycin (Kulkarni et al., 2014). Bacterial membrane is the major component of OMVs. Hence it appears that vesicles could bind the peptide antibiotics because of their affinity for membrane. On the other hand, the hydrophilic nature of streptomycin might be responsible for inability of the vesicles to bind it. Protective effect of the OMVs in *E. coli* against two membrane-active antibiotics (polymixin B and colistin) was evidenced earlier by the improved survival of a hypervesiculating mutant in presence of the antibiotics compared to the growth of the wild type strain challenged with the antibiotics. Vesiculation was found to be induced in the wild type strain in presence of the antibiotics and addition of purified vesicles to the wild type culture was also associated with improved survival in presence of the antibiotics. Similarly, survival of a strain of Enteropathogenic *E.coli* (ETEC) in presence of polymixin B could be improved by addition of vesicles produced by the strain to the culture medium (Manning and Kuehn, 2011).

Fluoroquinolones are widely used for the suppression of the mycoplasmas, which not only infect humans, animals, and plants but also contaminate tissue cultures and vaccines. Mycoplasmas rapidly develop resistance to the fluoroquinolone antibiotics. In a recent study, vesicles produced by a ciprofloxacin-resistant mutant of the mycoplasma *Acholeplasma laidlawii* were found to contain ciprofloxacin. Thus, the OMVs appeared to protect the bacterium from the antibiotic by removing it from the cell and not allowing it to accumulate in sufficient concentration required for its suppressive effect (Medvedeva et al., 2014). It was demonstrated earlier that production of vesicles in *E coli* was enhanced by increase in temperature and misfolded proteins, formed by heat stress, were packaged and removed from the cells by the vesicles (McBroom and Kuehn, 2007). Thus, OMVs appear to help the producer organism in getting rid of toxic and injurious substances by transporting them out of the cell.

Carbapenems are one of the last resorts for the clinical management of antibiotic-resistant pathogens. It was observed that the plasmid-borne *bla*_*OXA*−24_ gene, which encodes a β-lactamase, responsible for carbapenem-resistance of *Acinetobacter baumannii*, was present in the OMVs produced by two clinical isolates of the organism. Following incubation with these OMVs, a carbapenem-sensitive strain of the same bacterium was found to acquire resistance. Subsequently it was found to release OMVs containing the *bla*_*OXA*−24_ gene (Rumbo et al., 2011). Transfer of penicillinase-specifying R plasmids from antibiotic-resistant *Neisseria gonorrhoeae* to the wild-type cells was demonstrated earlier (Dorward et al., 1989). The vesicles produced by the mycoplasma in the above-mentioned example were also found to contain a mutant version of the gene that encodes ciprofloxacin-targeted protein (Medvedeva et al., 2014). Hence OMVs could assume the role of a vector in horizontal gene transfer that plays a crucial role in dissemination of antibiotic-resistance among natural isolates.

Bacteria in biofilms are known to be more resistant to antibiotics than their planktonic counterparts. A recent proteomic analysis of OMVs obtained from planktonic growth and biofilm in *Pseudomonas aeruginosa* revealed that drug-binding proteins (notably efflux proteins) were more concentrated in the biofilm OMVs. It is another example of possible involvement of the vesicles in antibiotic resistance (Park et al., 2014).

## Scope for further studies

The active involvement of OMVs in antibiotic-resistance of bacteria emphasizes the necessity of further in-depth investigations on this aspect. Isolation of clinical samples of antibiotic-resistant bacteria and analysis of the molecular profile of the vesicles produced by them are likely to promise insight into the molecular basis of the antibiotic-resistance. Search for the inhibitors of vesicle formation (Tashiro et al., 2010) appears to hold some promise in therapeutics since by suppressing vesicle formation; they are likely to suppress both pathogenesis and antibiotic-resistance of the pathogens.

### Conflict of interest statement

The authors declare that the research was conducted in the absence of any commercial or financial relationships that could be construed as a potential conflict of interest.
