# Corrigendum: Vesicles-mediated resistance to antibiotics in bacteria

**DOI:** 10.3389/fmicb.2015.00974

**Published:** 2015-09-10

**Authors:** Madhab K. Chattopadhyay, Medicharla V. Jagannadham

**Affiliations:** Centre for Cellular and Molecular Biology (CSIR)Hyderabad, India

**Keywords:** antibiotic-resistance, outer membrane vesicles, OMVs, β-lactamases, membrane-active antibiotics, fluoroquinolones, carbapenems, horizontal gene transfer

The name of the second author should be read as Medicharla V. Jagannadham.

The original article was updated.

## Conflict of interest statement

The authors declare that the research was conducted in the absence of any commercial or financial relationships that could be construed as a potential conflict of interest.

